# Accelerated 2D magnetic resonance spectroscopy of single spins using matrix completion

**DOI:** 10.1038/srep17728

**Published:** 2015-12-03

**Authors:** Jochen Scheuer, Alexander Stark, Matthias Kost, Martin B. Plenio, Boris Naydenov, Fedor Jelezko

**Affiliations:** 1 Institute for Quantum Optics and Center for Integrated Quantum Science and Technology (IQST), Albert-Einstein-Allee 11, Universität Ulm, 89069 Ulm, Germany; 2 Institute for Theoretical Physics and Center for Integrated Quantum Science and Technology (IQST), Albert-Einstein-Allee 11, Universität Ulm, 89069 Ulm, Germany

## Abstract

Two dimensional nuclear magnetic resonance (NMR) spectroscopy is one of the major tools for analysing the chemical structure of organic molecules and proteins. Despite its power, this technique requires long measurement times, which, particularly in the recently emerging diamond based single molecule NMR, limits its application to stable samples. Here we demonstrate a method which allows to obtain the spectrum by collecting only a small fraction of the experimental data. Our method is based on matrix completion which can recover the full spectral information from randomly sampled data points. We confirm experimentally the applicability of this technique by performing two dimensional electron spin echo envelope modulation (ESEEM) experiments on a two spin system consisting of a single nitrogen vacancy (NV) centre in diamond coupled to a single ^13^C nuclear spin. The signal to noise ratio of the recovered 2D spectrum is compared to the Fourier transform of randomly subsampled data, where we observe a strong suppression of the noise when the matrix completion algorithm is applied. We show that the peaks in the spectrum can be obtained with only 10% of the total number of the data points. We believe that our results reported here can find an application in all types of two dimensional spectroscopy, as long as the measured matrices have a low rank.

A key tool in the quest for the determination of the structure of molecules and proteins is nuclear magnetic resonance spectroscopy (NMR) which has helped to make fundamental contributions to the advancement of biological sciences. This is achieved by measuring the magnetic response of molecules in a large ensemble to sequences of radio frequency pulses. This temporal response is then mapped to multi-dimensional spectra which encode the dynamical properties of the system and therefore the interactions between its constituent nuclear spins^1^,^2^. The information contained in these spectra forms the basis for the determination of molecular structure. Current NMR schemes are intrinsically ensemble measurements, both due to the minute size of the nuclear magnetic moments and the tiny polarization of these nuclear spins at room temperature, even in very strong magnetic fields. Consequently, NMR can only deliver ensemble information while the structure and dynamics of individual specimens remain hidden from observation.

Recent progress in the control of the single electron spin in nitrogen-vacancy (NV) centers in diamond offers a new perspective here, as it can make use of optically detected magnetic resonance^3^,^4^ for the detection of material properties^5^ including minute magnetic fields^6^,^7^,^8^,^9^. Building on this, recent theoretical investigations^10^,^11^,^12^,^13^ have suggested that NV centers implanted a few nanometers below the surface should be able to detect and locate individual nuclear spins above the diamond surface. Subsequent experimental work has indeed achieved the observation of small clusters of nuclear spins outside of diamond with a sensitivity that is sufficient to identify even individual nuclear spins^14^.

One of the challenges for the determination of the structure of smaller biomolecules or even entire proteins by means of 2D spectroscopy detected by a NV center is the considerable amount of data that need to be taken which results in long measurement times. Indeed, the large amount of required data and the associated long measurement times represent a challenge that is common to both ensemble NMR and single molecule NMR measurements.

As suggested in^12^ we demonstrate NV sensing experiments on nuclear spins using methods from the field of signal processing, particularly matrix completion^15^,^16^. With this technique we can obtain reliably the spectral information that is contained in 2D-NMR spectra from a small subset of all accessible data points (see^17^,^18^ for applications of the related but distinct compressive sensing and non-uniform sampling to bulk NMR). The results presented here show that order of magnitude reduction in the overall measurement time in NV center based 2D-NMR can be achieved.

In the remainder we briefly introduce matrix completion in Section II. Then Section III presents the application of this method to concrete experimental data that have been obtained from a NV center interacting with a nearby nucleus. The results demonstrate that already a sampling rate of around 10% suffices to reconstruct the spectral information reliably. We finish with a brief conclusion and outlook concerning the potential of this approach for diamond quantum sensing.

## Matrix Completion Method

This section serves to introduce briefly the concept of matrix completion, the basic properties relevant to this work and the specific algorithm that we use for its application to our experimental data.

A 2D-spectrum encodes the response of a system to a sequence of pulses with varying temporal separation, denoted by 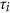 and 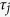, and the data is arranged in a matrix 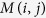. The 2D-spectrum 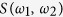 is then obtained as the Fourier transform of both time coordinates in *M*. In our work we are sampling randomly chosen elements of the matrix 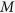 with indices 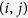 drawn from the index set 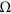, leading to constraints 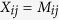 for 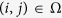. Matrix completion solves the task of obtaining the missing matrix entries of *M* that have not been measured in experiment. In general this is impossible unless we have further knowledge about the matrix 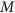, namely that it typically has a low singular value rank 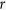, i.e. 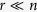 for the *n* × *n* matrix *M*.

One possible approach to achieve this matrix completion is by solving the minimization problem


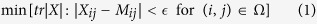


where 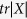 is the trace norm of the matrix *X* and 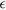 is a given tolerance. Indeed, it can be proven that this formulation of the problem achieves the desired aim^19^ as the solution of eq. (1) yields the matrix *M* with high probability if the number of sampled elements 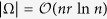 (see^19^,^20^ for proofs and a rigorous mathematical statement). This suggests that a computational gain by a factor of order 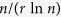 may be achieved through random sampling in the manner described above (see for example^12^,^21^ on computed 2D-spectroscopy data).

This still leaves us with the task of solving the minimization problem eq. (1). In principle, this equation can be rewritten as a semi-definite programme and then solved employing standard solvers for convex problems. Unfortunately, standard solvers tend to be limited to relatively small matrix sizes, but fortunately alternatives exist. Indeed^22^, proposed to solve eq. (1) approximately through the so-called singular value thresholding (SVT) algorithm^22^ which permits very large matrices to be treated. It is this algorithm that we will be using in our work. The SVT-algorithm solves iteratively the set of equations


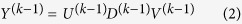



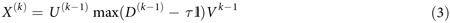



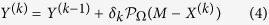


where 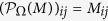 for 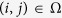 and zero otherwise and eq. (2) represents the singular value decomposition of the matrix 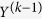. 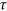 and 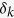 are free parameters in the procedure that regulate the soft thresholding (eq. 4) and the inclusion of the constraints (eq. 5). The choice 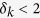 ensure provable convergence and 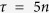 for *n* × *n*-matrices represent typical values (see^22^). As a termination criterion of the iteration we employ the condition


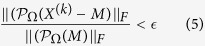


for some 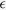, and 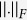 being the Frobenius norm^22^. The algorithm employs a singular value decomposition which, for large matrices, can be accelerated considerably^23^,^24^. It is also noteworthy that other approaches for solving eq. (1) such as those reported in^25^,^26^,^27^ may lead to improved performance and/or stability but for the purposes of this study SVT was sufficient and recommended itself thanks to its ease of implementation. Below we give a pseudo code which implements the matrix completion algorithm:

//matrix M subsampled on Ω//

Y = 0

while ε<ε_0_ do

*U*, *S*, *V* = *SVD*(*Y*)//singular value decomposition


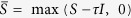
 //element wise thresholding


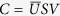



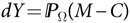



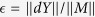


*Y* = *Y* + *δdY*

end do

return C//completed matrix//

In any real-world application, the measured entries of the data matrix will be corrupted at least by a small amount of noise. Hence the question of the robustness of the matrix completion approach to fluctuations in the experimental data arises naturally. Reassuringly, results have been developed that guarantee that reasonably accurate matrix completion is possible from noisy sampled entries^28^. In that scenario noise can be neglected if the relevant spectral information can be still extracted from the low rank approximation of *M*, thus implicating a sufficiently large signal-to-noise ratio and leading to the fact that noise contribution results in small singular values, which are discarded after applying our algorithm. Hence matrix completion offers three major advantages:

- Weak noise is directly suppressed by the matrix completion algorithm
- The spectrum of the system can be recovered from a small subset of all data e.g. only 10

% of the total in our examples.
- Usually compressed sensing requires some additional information in order to effectively recover the measured data, e.g. sparse basis^29^, although a random basis under broad assumptions can be also used. The only requirement for matrix completion in our application (where sampling is carried out in Fourier space) is that the matrix with the data needs to have a low rank.

The following section will now present the result of the application of the matrix completion algorithm to concrete experimental data that have been obtained in our laboratory.

## Experimental Implementation

### 2D ESEEM with a single NV centre

The method of matrix completion has been implemented in 2D optical spectroscopy of Rb vapour^30^. We use a single NV centre in diamond coupled to a proximal ^13^C nuclear spin as a test system for the demonstration of the matrix completion protocol. NVs are optically active point defect centres in the diamond crystal. Their fluorescence depends on the electron spin number 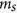 of the triplet ground state, allowing to measure the electron spin of single centres. NVs close to the diamond surface have been used to detect few thousand external protons^31^,^32^ followed later by a demonstration of even single spins sensitivity^14^ leading to nano-scale magnetic resonance imaging^33^,^34^,^35^. For these types of experiments the data acquisition is quite long due to the low fluorescence emission from single centres.

**Figure 1:**
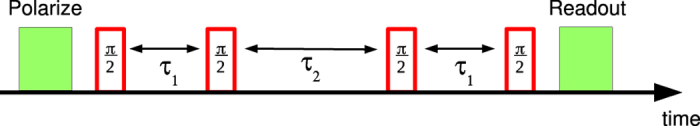
Pulse sequence for the two dimensional ESEEM measurement used in our experiments. See text for detailed description.

The NV has a triplet ground state (electron spin *S* = 1) coupled to the nitrogen nuclear spin (^14^N, *I* = 1). The system can be described by the Hamiltonian:


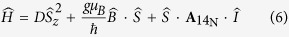


where 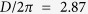 GHz is the zero field splitting of the ground state, 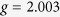 is the Landé factor, 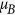 is the Bohr magneton, 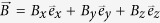 is the applied static magnetic field, 
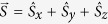
 and 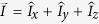 are the electron and nuclear spin operators and 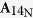 is the hyperfine interaction tensor. The *z* axis is taken to be along the NV crystal axis. If there is a single ^13^C nuclear spin (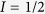) in the proximity, the following term


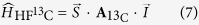


is added to the spin Hamiltonian 6, with **A**_^13^C_ being the hyperfine interaction tensor to a ^13^C nuclear spin. One of the simplest 2D NMR experiments consists of three 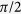 pulses and is called correlation spectroscopy (COSY)^36^. In our work we use its “equivalent” in the electron spin resonance-the three pulse electron spin echo envelope modulation (ESEEM) pulse sequence (also called stimulated echo, see^37^ for more details) shown in Fig. 1.

The sequence starts with a laser pulse of about 3 *μ*s to polarize the NV electron spin in the 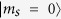 state. Afterwards we apply four 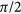 microwave pulses at times *t* = 0, 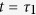, 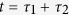 and 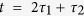. The last pulse is used to transfer the electron spin coherence into population, which is read out by the last laser pulse. The spin signal is recorded for each pair of (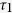, 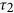) and then a 2D Fourier transform is performed giving a set of frequencies (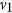, 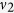). From this spectrum the number of nuclei coupled to the electron spin and the off diagonal elements of the hyperfine interaction tensor (e.g. proportional to 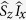) can be obtained^37^.

We applied this pulse sequence in two different experiments. In the first measurement we use a single NV without resolvable coupling to ^13^C spins. The system consists of a NV electron and a nitrogen nuclear spin, which are described by the Hamiltonian in 6. If the static magnetic field is aligned with the NV axis, the hyperfine interaction tensor 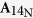 is diagonal and there is no ESEEM effect. In order to introduce artificial “off-diagonal” terms, we apply the static 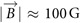 off-axis, at an angle of about 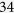 degrees with respect to the *z* axis. The expected 2D spectrum 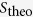 can be simulated by using the Hamiltonian 6 and is plotted in 2a.

**Figure 2:**
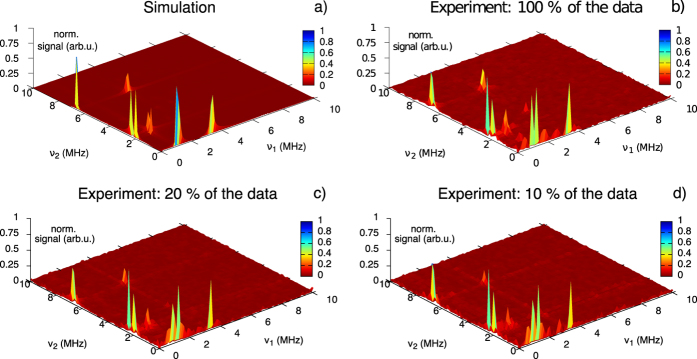
2D ESEEM simulation and experimental data with a single NV when static magnetic field 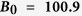 G is applied at an angle of 34.1°. (**a**) Simulation using the Hamiltonian (6). (**b**) Fourier transform of the complete set of the experimental data points 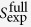. Fourier transform of the experimental data after applying matrix completion and using 20% 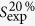 (**c**) and 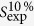 10% (**d**) of the time domain data. The main peaks are still observed even when 90% of the data is removed.

In Fig. 2b we plot the Fourier transform of the experimental data 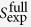, where we use all collected data points. The experiment agrees well with the simulation. In order to demonstrate the performance of the matrix completion method, we use a random mask Ω to remove a certain part from the full experimental data in the time domain. After that, we apply matrix completion using the SVT algorithm as described in Section Matrix Completion Method to recover the full matrix. A Fourier transform of the matrix obtained with 20% of the initial data points 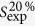 is shown in Fig. 2c. Despite the removal of 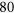 % of the recorded data, the number of peaks and their positions are still present if we compare to Fig. 2b. Even if we keep only 10% of the original matrix (see Fig. 2d), we can still recover the relevant spectral information.

**Figure 3:**
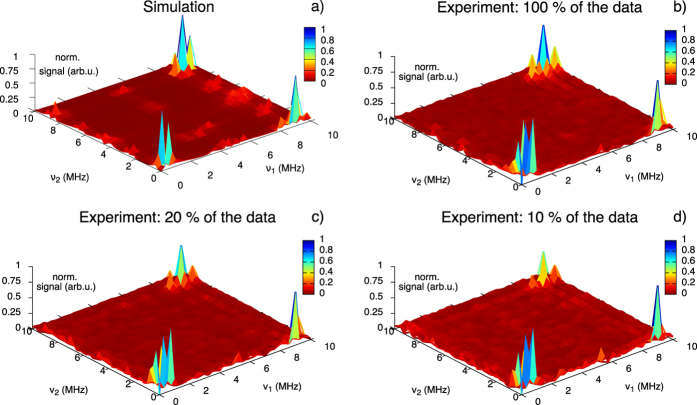
2D ESEEM simulation and experimental data with a single NV coupled to a single ^13^C nuclear spin. (**a**) Simulation using the spin Hamiltonian (6) and (7). (**b**) Fourier transform of the complete set of the experimental data points. Fourier transform of 20% (**c**) and 10% (**d**) of the data in the time domain. Here we again can recover the spectral information by keeping small amount of the experimental data.

In the second experiment we localized a NV coupled to a single ^13^C spin with a coupling strength of 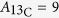 MHz. Now, depending on the position of this carbon atom with respect to the NV, there are different hyperfine interaction tensors^38^,^39^,^40^. The spectrum can be calculated by using the Hamiltonian 6 and 7 by choosing the correct hyperfine interaction tensor. The simulation is shown in Fig. 3a.

In these measurements the magnetic field has been aligned along the NV axis. The 2D Fourier transform of the full data set is shown in Fig. 3b. As in the previous experiment, we can still recover the full spectral information (cf. Fig. 3c), if we remove randomly 80% of the data points. From Fig. 3d we can conclude that even 10% of the data suffice for the matrix completion algorithm to obtain the spectrum and the hyperfine coupling strengths are still well resolvable. If we keep even less data points, the spectrum becomes distorted (data not shown). In fact, this factor of ten is what is expected from the theory, see Section Matrix Completion Method and below.

### Performance of the matrix completion algorithm

**Figure 4:**
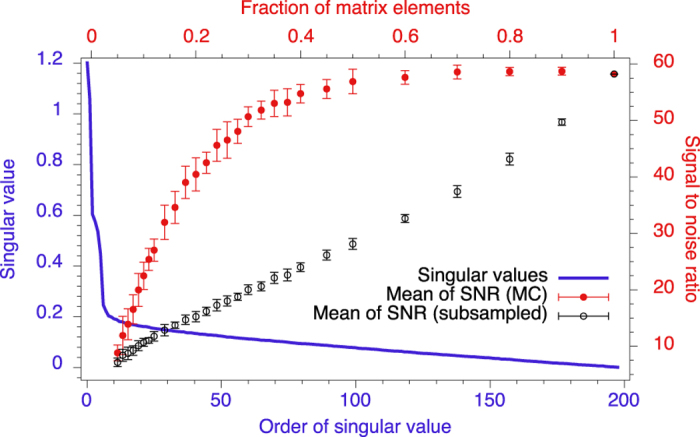
(**a**) SNR of the 2D spectrum when the data is recovered with the matrix completion algorithm (red markers, right axis) and randomly subsampled matrix (black markers, right axis) as a function of the fraction of sampled elements (top axis) taken from the experimental data shown in Fig. 3. The matrix completion algorithm and subsampling were performed at least 10 times, here the errorbars denote the standard deviation. The blue curve (left axis) represents the singular values 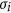 of the measured data (Fig. 3b) in descending order.

In the following the performance of the matrix completion algorithm will be analysed on our experimental data. For this purpose we first quantify how the signal to noise ratio (SNR) of the 2D spectrum depends on the number of matrix elements remaining from the complete data set. We use the experimental data shown in Fig. 3. In Fig. 4 the results of this analysis is plotted. In order to calculate the SNR we take the maximal signal of the (9 MHz, 9 MHz)-peak and compare it to the mean of a region which contains only noise.

We compare the SNR of two methods to obtain the spectrum. With the first we applied the matrix completion algorithm to recover the matrix in the time domain and then we perform a Fourier transform (red markers). With the second we set randomly chosen matrix elements to zero and then again transform it in the frequency domain (subsampled, black markers). In the latter case the SNR drops almost linearly when the fraction of known matrix elements decreases. When the matrix completion is used the SNR remains almost constant until roughly 40% of the matrix elements are retained, by which time it starts to drop, but it remains significantly larger than the SNR obtained for subsampled data. In the same plot we give the ordered singular values 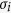 of the measured matrix, showing that about 70% of them are close to zero, suggesting that they contain predominantly noise.

In the following we define a fidelity which is a measure of how well we can recover the complete matrix if we use a fraction of its elements. Here we used measurement data with very low noise in order to have an ideal data set. The matrix 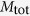 which contains all the measured data and the matrix 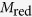 which is obtained from subsampling either without further processing (subsampled) or after application of the matrix completion algorithm (MC). By using these two matrices, we define the fidelity *F* of our algorithm as


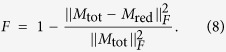


**Figure 5:**
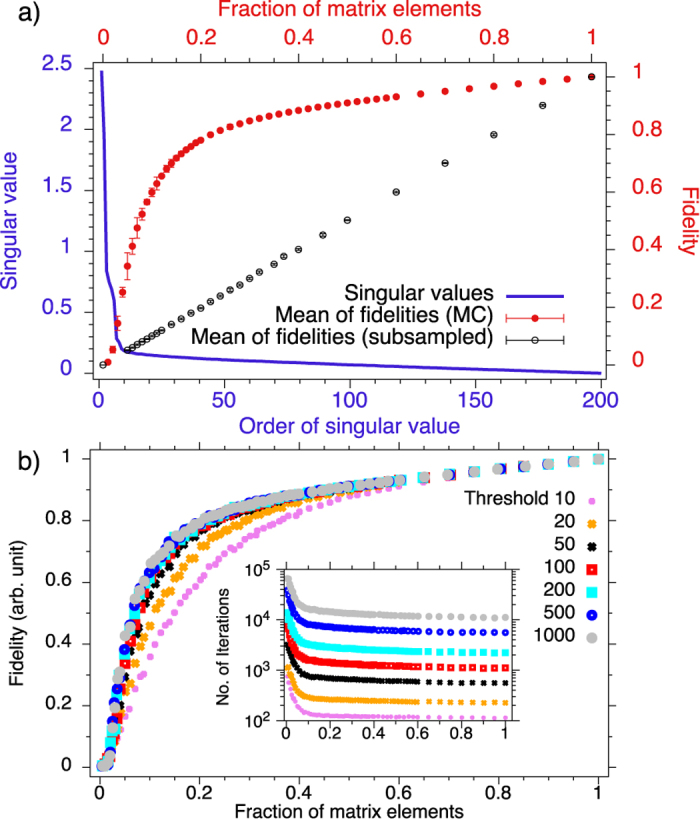
(**a**) Mean of fidelities of the matrix completion algorithm (red markers, right axis with 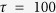) and randomly subsampled matrix (black markers, right axis) as a function of the fraction of sampled elements (top axis). The matrix completion algorithm was performed 128 times with each time different random sampling, here the errorbars denote the standard deviation. The blue curve (left axis) represents the singular values 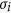 of the measured data in descending order. (**b**) The fidelity of the matrix completion algorithm as a function of the fraction of matrix elements at different thresholds 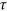. Inset: Number of iterations required to run the matrix completion algorithm as a function of the threshold and the fraction of the matrix elements.

In Fig. 5a we plot the fidelity as a function of the fraction of the elements of the complete matrix (red markers).

In the same plot we show the ordered singular values 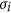 of the matrix with the full number of points 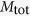. Additionally we show the fidelity when we randomly set a fraction of the matrix elements to zero. In the latter case *F* decreases linearly as expected. From the plot we can conclude that the matrix in the time domain can be recovered by using 10% of the elements of 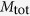, since only these elements are significantly larger than zero. This result is consistent with the theoretical limit for recovering 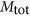 given by only 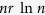
19,20 matrix elements (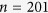) where we can roughly assume a rank 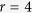, which is the number of the peaks in the spectrum. It is interesting to investigate the influence of the threshold parameter 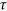 (see eqs 2, 3) on the performance of the SVT algorithm and the fidelity of the so determined spectra (see Fig. 5b). Too small threshold values 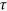, e.g. at 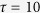 or 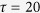 (pink and orange markers), lead to low fidelity when less than 60% of the matrix elements are sampled. We can achieve higher fidelities 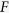 by increasing 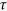 and we observe saturation around 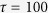. That is, for 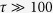 we cannot obtain higher significantly higher fidelities, while the required computation time (equivalent to the number of iterations) increases which can be seen in the inset graph in Fig. 5b. From there we can conclude that with our data set thresholds even below the empirically suggested rule 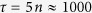 for our case of 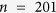 (see Section Matrix Completion Method and^22^) are sufficient. A python script can be found in the supplementary material, where the SVT algorithm is implemented, together with an experimental data set.

## Conclusions

In summary, we have demonstrated the application of a method for reconstructing a two dimensional ESEEM spectrum, by collecting only small part of the data in the time domain. With our technique we can obtain the necessary spectral information by measuring 10% of the experimental data points in two different experiments. By using our method, the measurement time can be shortened by a factor 10 compared to the conventional experiment. We believe that the reported results will be useful for any type of 2D NMR and ESR spectroscopy and also for magnetic resonance imaging. Our method is particularly useful for single spins experiments, which usually require very long measurement times^13^,^35^.

## Methods

The diamond samples having NV centres created during the chemical vapour deposition (CVD) process (CVD) have been provided by Element 6, Ltd. All experiments have been performed on a home built confocal microscope. For the microwave manipulation we used a continuous wave MW source (Rohde und Schwarz, SMIQ03B), a MW switch (Mini circuits, ZASWA-2-50DR+) and a pulse generator to form MW pulses (Tektronix, AWG7122C). The constant magnetic field has been provided by a permanent magnet mounted on a computer controlled 3D stage equipped with a rotational module (Micos LS110, PR110).

The matrix completion algorithm has been implemented using the Python language. The program for simulating the 2D spectra has been written in Fortran. All calculation have been performed on a standard desktop computer.

## Additional Information

**How to cite this article**: Scheuer, J. *et al.* Accelerated 2D magnetic resonance spectroscopy of single spins using matrix completion. *Sci. Rep.* **5**, 17728; doi: 10.1038/srep17728 (2015).

## Supplementary Material

Supplementary Dataset
